# A Practical Treatise on Healthy Skin: With Rules for the Medical and Domestic Treatment of Cutaneous Diseases

**Published:** 1846-01

**Authors:** 


### ART. XVII.

*A practical Treatise on Healthy Skin: with Rules for the Medical and Domestic Treatment of Cutaneous Diseases.* By ERASMUS WILSON, F.R.S., Lecturer on Anatomy and Physiology in the Middlesex Hospital, &c. &c. Illustrated with Six Steel Engravings.—*London,* 1845. Small 8vo, pp. 356.

"IN the following pages," commences our author, "I propose to make my reader acquainted with the structure and uses of the skin, in the hope of awakening his attention to the necessity and manner of training it to the purposes of health. I trust, moreover, by laying down correct and simple laws, to enable him to comprehend the principles upon which a sound and effective domestic treatment of its diseases may be conducted." We must confess ourselves to be rather at a loss to know, whether Mr. Wilson's objects be to address the general public alone, or whether he aims at including his professional brethren among his readers. If the former, we must say that we cannot find sufficient reason for selecting an isolated portion of general hygiène, treating it at such length and with such copiousness, and especially for appending to it so much information of a purely medical kind, though conveyed in language anything but professional;—except in those unworthy motives, which we have always felt it our duty to expose and condemn, from which we believe Mr. Wilson to be altogether exempt. If the latter, as would appear from the details here and there interspersed on some departments to which Mr. Wilson has devoted peculiar attention, as well as from the combination of rules for the medical treatment, with those for the domestic management, of cutaneous diseases, we think that Mr. Wilson would have done much better to put his Exposition into a more professional form. We believe that there are many amongst his seniors, as well as his juniors, who would have gladly welcomed such a work, embracing the results of the continued attention which he has given to the subject, since the publication of his more formal Treatise, and devoted rather to the prophylaxis and the general management, than to the classification and particular treatment; of these ill-understood and most troublesome maladies.

The general management of the skin has been ably treated by many previous writers on popular hygiène; and particularly by Dr. Andrew Combe, whose chapter on this subject contains almost every direction of real importance. If the brain, the lungs, the heart, the stomach, the liver, the kidneys, &c. &c., were each treated in a separate volume, with the copiousness of illustration which Mr. Wilson has bestowed on his favorite organ, the Skin, the public would be in possession of a complete encyclopædical library of hygiène, which would not, we apprehend, be nearly as useful as Dr. A. Combe's comprehensive treatise; on account of both the expense and the time required for acquaintance with its contents. In some respects, however, the present publication is well timed: for it may serve to forward the "bath and wash-house" movement; which, combined with those which are taking place of early shop-closing, of draining and ventilation, and of public places of recreation, we look upon as among the most pleasing and favorable of the "signs of the times." If such be Mr. Wilson's purpose, we are ready to accept his contribution to the general impulse that is now operating so beneficially on the public mind, as one of no mean value, but we would have been better pleased if he had given a more prominent expression of his intentions, and had omitted many passages that appear to us to have somewhat of a too popular bearing.

We shall now extract, as specimens of the mode in which the different subjects are handled, a few passages which may, at the same time, communicate useful information to our readers. After a chapter on the scarf-skin, and another on the true skin, which contain clear and accurate descriptions of those structures, couched in popular language, our author proceeds to the perspiratory system; which he has investigated with particular minuteness.

"Taken separately, the little perspiratory tube, with its appended gland, is calculated to awaken in the mind very little idea of the importance of the system to which it belongs; but when the vast numbers of similar organs composing this system are considered, we are led to form some notion, however imperfect, of their probable influence on the health and comfort of the individual. I use the words 'imperfect notion' advisedly; for the reality surpasses imagination, and almost belief. To arrive at something like an estimate of the value of the perspiratory system, in relation to the rest of the organism, I counted the perspiratory pores on the palm of the hand, and found 3528 in a square inch. Now, each of these pores being the aperture of a little tube of about a quarter of an inch long, it follows that in a square inch of skin on the palm of the hand, there exists a length of tube equal to 882 inches, or 73½ feet. Surely such an amount of drainage as 73 feet in every square inch of skin, assuming this to be the average for the whole body, is something wonderful; and the thought naturally intrudes itself, What if this drainage were obstructed? Could we need a stronger argument for enforcing the necessity of attention to the skin? On the pulps (?) of the fingers, where the ridges of the sensitive layer of the true skin are somewhat finer than in the palm of the hand, the number of pores on a square inch a little exceeded that of the palm; and on the heel, where the ridges are coarser, the number of pores on the square inch was 2268, and the length of tube 567 inches, or 47 feet. To obtain an estimate of the length of tube of the perspiratory system of the whole surface of the body, I think that 2800 might be taken as a fair average of the number of pores in the square inch; and 700, consequently, of the number of inches in length. Now, *the number of square inches of surface in a man of ordinary height and bulk is* 2500; *the number of pores, therefore,* 7,000,000, *and the number of inches of perspiratory tube* 1,750,000, *that is,* 145,833 *feet, or* 48,600 *yards, or nearly* 28 *miles.* [Note. To the medical reader it may be necessary to explain, that the sebaceous system is included in the system of perspiratory glands and tubes in this calculation. I have ascertained beyond question, that the sebaceous system is the perspiratory apparatus of the greater part of the body, the true perspiratory glands and tubes being found only in certain parts. Therefore, the calculation which I have made on these premises must be considered as falling within rather than beyond the truth."] (p. 43.)

The next chapter, "On the oil-glands of the skin," contains an account of the sebaceous follicles, which differs in some degree from that which is generally received, and which is probably more accurate.

"The apparatus for keeping the surface of the skin bedewed with an oily fluid, resembles, in general particulars of structure and economy, that of the perspiratory system. It consists of minute tubes, which traverse the scarf and sensitive skin, and enter the substance of the corium, where they terminate in small glands. These tubes are similar in structure to the perspiratory ducts, being composed of three layers, derived respectively from the scarf-skin which lines their interior; the sensitive skin, which is the medium of distribution of their vessels and nerves; and the corium, with its fibres, which gives them strength and support. Like the perspiratory tubes, they are in some situations spiral, but this is not a constant feature; more frequently they pass directly to their destination, and they are also larger. The chief characters in which they differ from the perspiratory apparatus are, the straightness and greater diameter of their tubes, their absence in certain situations, as on the palm and sole, and abundance in others where their office is more needful, as on the face and nose, the head, the ears, &c., and the degree of complication in the structure of their glands. This latter character is sufficiently remarkable, since they offer every shade of complication, from the simple straight tube, to a tube divided into numberless ramifications, and constituting a little rounded arborescent mass, of about the size of a millet seed." (p. 56.)

Mr. Wilson considers that the healthy action of the sebaceous system is peculiarly interfered with by the "sedentary and irregular habits of refined society." Instead of being completed according to the normal standard, the secreting operations are irregular and torpid, the contents of the cells unnaturally solid and dense; they are only partly or not at all emptied, and are either thrown out in a mass upon the surface of the skin, or, if too dense and dry for this mode of escape, they collect in the tube of the gland, and produce an unnatural distension of it, which frequently gives rise to inflammation. It is in this condition of the sebaceous system, that the curious parasite, first discovered by Dr. Simon of Berlin, and subsequently investigated by Mr. Wilson, appears most disposed to lodgment and multiplication within the oil-tubes.*

* We cannot help noticing Mr. Wilson's defence of the title, which he has bestowed upon this animal, as showing a degree of disregard for the rules of zoological nomenclature, which we should scarcely have expected from him. Having designated it as the *Entozoon folliculorum*, and having

"The animalcule of the skin is found in the oil-tubes, whenever there exists any disposition to the unnatural accumulation of their contents; it is found in numbers, varying from one or two to twenty, in the interior of the little grooved cylinder, which is squeezed out by the pressure of the fingers; and this in an apparently perfect state of the health of the skin, cannot be said to be in perfect health when its functions are performed in a torpid manner. Now, as in the majority of mankind, and certainly in all the inhabitants of cities and large towns, the skin is more or less torpid in its functions, so the presence of this animal in the skin is the rule, its absence the exception. I have found it in all ages, from youth to old age, more numerously, it is true, at the latter than the former period, and in great and remarkable numbers during sickness. Under these circumstances I see no other conclusion open, than to assume that it performs some beneficent purpose in the economy of the skin; that purpose being, according to my belief, the disintegration of the over-distended cells, the impression of a new condition upon the contents of the cells, and the stimulation of their tubes to perform their office more efficiently. In corroboration of this view is the fact, that these little creatures increase in number when the vital powers decline, so that when the energies of the system are reduced by disease, and when the skin, participating in that reduction, is unable alone to fulfil its functions correctly, these little beings are produced to aid it in the work." (p. 62.)

This is certainly a somewhat favorable view of the purposes of this extensive parasitism; but we see no sufficient evidence in its favour; since the mere fact of multiplication of individuals under circumstances favorable to their existence, indicates nothing as to the final cause of their production. Doubtless it will be highly gratifying and instructive to the public mind, to be made acquainted with the fact, that the habits of "refined society" tend just as strongly to the production of this tribe of parasites, as the habits of the "great unwashed" favour the development of certain others, which are not in themselves one whit more disgusting. Here, as elsewhere, we see how nature manages to compensate one class for the exemption they may enjoy from the evils incident to another. The delicate town-bred lady of fashion, in descending from her carriage, shrinks instinctively from the mass of rags, filth, and vermin, which is brought referred to the fact that the word entozoon is now used to distinguish a *class* of animals, he nevertheless is bold enough to add, "I do not think this an objection to its use as a generic appellation; and the specific name which follows renders any mistake impossible." (p. 61, note.) Such a method of nomenclature, carried to its legitimate extent, would produce inextricable confusion, and endless absurdities. Fancy our calling the window-swallow *Avis fenestrarum;* or the bed-bug *Insectum lectularium.* In Mr. Wilson's detailed account of the animal, published in the Philosophical Transactions for 1844, the generic name *Entozoon* was only suggested as provisional; the class, to which the animal should be referred, being still a matter of question. We should not have noticed the subject here, had it not been that the error of nomenclature is not only continued, but defended, by Mr. Wilson. And the error is the greater, because it is acknowledged on all hands, that the animal is *not* one of the class entozoa, being raised by its complexity of structure far above that group; so that it is very much as though Mr. Wilson were to christen a bat by the title of *Avis speluncarum,* because it flies like a bird, and lives in caves. The fact is, that the parasite in question is one of those animals which sometimes present themselves, to the perplexity of the systematic zoologist, but to the satisfaction of the philosophical student of nature who regards classes, orders, &c., as a machinery of human invention, adapted to serve the purpose of facilitating the acquisition of knowledge, but as having no real existence in creation. It unites the characters of the higher annelida or worm-tribe, of the lower arachnida or spider-tribe, and of the lower crustacea or crab-tribe, to such a degree, that it is yet doubtful which class ought to afford it a place. Its structure and history have been so ably elucidated by Mr. Wilson in the paper we have referred to, that little probably remains to be added to the information he has collected; and the difficulty in assigning its zoological position lies rather in our imperfect acquaintance with the nearest-allied forms among the classes just named. The investigator of the history of this parasite need certainly be in no lack of *subjects* for examination; for, strange to say, it is one of the most readily procurable of all living beings, especially in a town population.

into contiguity with her precious person by some pertinacious beggar; ignorant all the while that her sebaceous follicles give board and lodging to a host of parasites, whose number may equal that of the various kinds of "small deer" that nestle in the matted hair and tattered garments of the fellow-being whom she regards with such loathing. "Where ignorance is bliss, 'tis folly to be wise;" and some, perhaps, of Mr. Wilson's fair readers may not thank him for enlightening them on the subject. But we consider that such knowledge ought not to be withheld from deference to fastidious delicacy; and that, besides the immediate inducement it affords to the adoption of such habits as may free the sebaceous system of those unwelcome inhabitants, it may also have the beneficial *moral* use, of aiding in the demolition of those barriers between the classes of society on which we are thankful that every day is now making some fresh inroads.

The structure and development of the hairs, constitute the next subject of discussion; and these are described in accordance with the most recent microscopical observations. We cannot but think, however, that the comparison of the structure of the human hair with that of other animals might have been carried out a little farther, with much interest to the reader; and that it might have advantageously superseded the detailed account, than which nothing could possibly (in our estimation) have been more dry and purposeless, of the direction or *set* of the hairs on different parts of the body, occupying between *five* and *six* pages. This variety in the direction of the hairs can only be well observed in the down of the new-born infant; as the hairs soon cease to be developed on the greater portion of the surface, except in occasional instances. This down must be regarded as a rudimentary condition of the complete hairy covering of the lower mammalia, and particularly of the monkey tribe, in which the *set* of the hairs is obviously a matter of consequence, even serving in one instance to distinguish species that are otherwise nearly allied. But in the human species it cannot be regarded as of the least import; as the character disappears so early, and is restricted to hairs of such minuteness. The following observation is new, so far as we know, and of some interest.

"The short hairs of the skin are not unfrequently disturbed in their growth by a cause previously referred to in connexion with the hairs of the head; namely, deficient oleaginous qualities of the product of the oil-tubes. When this happens, the dry scales and cells of the oil and hair tubes collect at the aperture of the latter, and become a source of impediment of growth to the hair, which, as a consequence, assumes a coiled and twisted appearance. But sometimes this closure of the hair-pore takes place after the fall of the old, and previously to the growth of the young hair, in which case the latter is imprisoned in the tube, and there grows, although unable to escape. I have occasionally seen nearly the whole of the hairs of a limb thus imprisoned, and forming little spiral circles, (can such things be?) which are visible through the then horny scale of desiccated cells, which covers them, and keeps them down. The obstruction occasions, as may be anticipated, a good deal of itching and uneasy feeling in the skin, and is more or less alleviated by tearing up the filmy covering." (p. 80.)

The itching of the dry scalp is referred by Mr. Wilson to a similar cause; the hair not being positively kept in, but being retarded, by the accumulation of the hard unctuous matter poured into the tube; and being thus caused to make pressure on the bulb at its base, which is supplied with sensory nerves. "A natural remedy for the unpleasant sensation is at hand; the nail is conveyed to the seat of inconvenience, it disturbs the impacted matter at the aperture of the tube, probably dislodges it, and the hair resumes its accustomed state." Prevention is better than cure, however, in this and other cases; and the proper use of soap and towel, comb and brush, will generally suffice.

It seems from the following observations that we have more hairs about our persons than we are commonly aware of:

"The invisible or downy hairs of the body rarely appear above the level of the skin, for when they do they necessarily fall into the category of the short hairs, and may best illustrate them by referring to a position in which their presence is invariable, although seen in that situation as an exceptional occurrence: I allude to the nose. The nose is ordinarily bald; but if the unctuous product of the oil-gland be squeezed out of its tube, and examined beneath the microscope, one or more of these little hairs will constantly be detected in the centre of the mass. Indeed, when the unctuous matter has been detained in the oil-tube for any length of time, the number of hairs may be considerable, as, for example, twenty, thirty, or even forty. Now, the whole of these hairs have, as far as we at present know, been produced by a single pulp, and having attained maturity, have been shed, to be carried out of the oil-tube with the unctuous substance; but the latter being retained, they have had time to collect, as we have seen, in astonishing numbers. I should be inclined to infer, from this circumstance, that these little hairs grow very quickly, and are shed at short intervals of time. On no other hypothesis can their numbers be satisfactorily explained. In their normal state and position, these little hairs are colourless and transparent, having rounded blunt points and brush-like roots; but under the influence of augmented action of the skin, they are susceptible of growth to a considerable extent, both in length and bulk, in fact, of becoming equal in dimensions to the short hairs of the body. Of this we have an example in the occasional growth of visible hairs upon the nose." (p. 82.)

Mr. Wilson expresses himself with great incredulity as to the rapid change in the colour of the hair, which has been alleged to take place not unfrequently, especially under the influence of strong mental emotion; and not content with expressing his entire discredit of the statements on record, as to the occurrence of such a change in a single *night*, he does not admit that even in a whole *week* anything more can take place than a whitening at the roots of the hairs. In fact, he considers it impossible, that the part of the hair which has once emerged from the hair tube can be affected by any subsequent changes in the condition of the body. We must confess that we cannot share with him in this incredulity; since there is nothing to our minds so imposssible, in the transmission of a chemical agent capable of effecting the change, from the base to the tip of the hair. What we know of the nutrition of other extra-vascular tissues fully warrants this idea; and it is further sanctioned by the fact, that the change in the colour of the entire fur, which takes place in some animals under the influence of cold, may be effected, under peculiar circumstances, within the time specified by Mr. Wilson. Thus a lemming, caught in the summer during one of the Arctic voyages, and kept in the cabin of a ship at a warm temperature, retained its summer coat far beyond the usual time; but when taken on deck on the 1st of February, and exposed to the intense cold of 30° below zero, the colour of its fur began to change in a few hours, and it was turned to a pure white in the course of a week. Now as we have no reason to believe that the direct influence of cold upon the pigmentary matter of the hair could produce any such effect, we must attribnte the change to some chemical influence propagated from the bulb. It would be interesting, however, for the sake of obtaining satisfactory proof of this position, to repeat this experiment, with the additional test of exposing a small quantity of hair, cut off before the animal had been subjected to a change of temperature, to the same degree of cold. If this should remain unaffected in hue, it is evident that the alteration must really take its origin in the growing point of the hair.

We cannot regard Mr. Wilson as justified in expressing his complete incredulity of the statement of Bichât, that he has seen hairs growing from mucous membranes. We cannot regard the assertion as by any means destitute of *à priori* probability, the relation of the skin and mucous membrane being kept in view; and we see no reason why the evidence of an observer so well qualified as Bichât should be set aside so unceremoniously.

The chapters on the maintenance of the health of the skin almost constitute a complete system of hygiène; the precepts given in regard to diet, exercise, clothing, &c., being just such as would lead to the maintenance of the health of the body in general, and therefore that of every one of its component organs. Therefore, the author's warnings aginst over-feeding, against the injurious practice of "converting the inside into a medicine-chest," with the view of expelling one evil by the introduction of another; against excessive, improper, or insufficient clothing; against excessive or insufficient exercise, &c. &c., all tell as much on the health of the stomach, the liver, &c., and numerous other organs, as on that of the skin. This we consider to be one of the unfavorable results of the isolation of this subject from general hygiène. The recommendations in regard to ablution as a prophylactic, and to the more systematic use of water in the cure of disease, fairly belong, however, to this department of the special hygiène. Mr. Wilson's remarks on these subjects are written with force and spirit; and his chapter on hydropathy contains what appears to us a fairer view of the merits of that method of treatment, than we have elsewhere seen from the pen of a regular medical practitioner. The following is its commencement:

"The advantages to health of a judicious and sound system of diet, clothing, exercise, and ablution, cannot be better illustrated than by reference to what has been termed 'the water-cure.' The water-practice has effected important results in the treatment of disease, and will, I trust, be instrumental in restoring to medicine one of her most valuable and important auxiliaries. Medical men may be jealous that these benefits have been 'conjured from the vasty deep' by other hands than those of the high-priests of Therapeia, but they have no just reason of complaint; the treatment of disease by water had been improperly neglected; now, however, its merits may be tested, and the test, aided by public encouragement; moreover, the remedy will revert to those who are alone qualified to employ it; and we may fairly hope that a correct system for its use will be established by their labours." (p. 136.)

The remaining part of the volume does not fall within *our* province. When we discuss medical topics, we like to call things by their scientific names; and we do not consider ourselves bound to take cognizance of the many pages of Mr. Wilson's book headed by St. Anthony's Fire, Mattery Pimples, Branny Tetters, and other old women's nomenclature. Any rules for the general management of skin diseases would be quite in keeping with our sense of propriety; but to encourage the public in the belief, that the treatment of erysipelas or rupia, to say nothing of impetigo and lepra, may be undertaken by unqualified domestic practitioners, appears to us a very injudicious, not to say dangerous measure.

When we next meet with Mr. Wilson we hope that it will be upon less doubtful ground. We shall with pleasure welcome him back to his own proper path,—that of accurate observation and clear description.

